# Metalloproteinase and inhibitor expression profiling of resorbing cartilage reveals pro-collagenase activation as a critical step for collagenolysis

**DOI:** 10.1186/ar2034

**Published:** 2006-08-18

**Authors:** Jennifer M Milner, Andrew D Rowan, Tim E Cawston, David A Young

**Affiliations:** 1 Musculoskeletal Research Group, 4th Floor Cookson Building, Medical School, Newcastle University, Newcastle upon Tyne, NE2 4HH, UK

## Abstract

Excess proteolysis of the extracellular matrix (ECM) of articular cartilage is a key characteristic of arthritis. The main enzymes involved belong to the metalloproteinase family, specifically the matrix metalloproteinases (MMPs) and a group of proteinases with a disintegrin and metalloproteinase domain with thrombospondin motifs (ADAMTS). Chondrocytes are the only cell type embedded in the cartilage ECM, and cell-matrix interactions can influence gene expression and cell behaviour. Thus, although the use of monolayer cultures can be informative, it is essential to study chondrocytes encapsulated within their native environment, cartilage, to fully assess cellular responses. The aim of this study was to profile the temporal gene expression of metalloproteinases and their endogenous inhibitors, the tissue inhibitors of metalloproteinases (TIMPs), reversion-inducing cysteine-rich protein with Kazal motifs (RECK), and α_2_-macroglobulin (α_2_M), in actively resorbing cartilage. The addition of the pro-inflammatory cytokine combination of interleukin-1 (IL-1) + oncostatin M (OSM) to bovine nasal cartilage induces the synthesis and subsequent activation of pro-metalloproteinases, leading to cartilage resorption. We show that IL-1+OSM upregulated the expression of *MMP-1*, -*2*, -*3*, -*9*, *12*, -*13*, -*14*, *TIMP-1*, and *ADAMTS-4*, -*5*, and -*9*. Differences in basal expression and the magnitude of induction were observed, whilst there was no significant modulation of *TIMP-2*, -*3*, *RECK*, or *ADAMTS-15 *gene expression. IL-1+OSM downregulated *MMP-16*,*TIMP-4*, and α_2_*M *expression. All IL-1+OSM-induced metalloproteinases showed marked upregulation early in the culture period, whilst inhibitor expression was reduced throughout the stimulation period such that metalloproteinase production would be in excess of inhibitors. Moreover, although pro-collagenases were upregulated and synthesized early (by day 5), collagenolysis became apparent later with the presence of active collagenases (day 10) when inhibitor levels were low. These findings indicate that the activation cascades for pro-collagenases are delayed relative to collagenase expression, further confirm the coordinated regulation of metalloproteinases in actively resorbing cartilage, and support the use of bovine nasal cartilage as a model system to study the mechanisms that promote cartilage degradation.

## Introduction

Articular cartilage is composed of one cell type, the chondrocyte [1], which is embedded within an extracellular matrix (ECM) of predominantly type II collagen and aggrecan (a large aggregating proteoglycan). A type II collagen scaffold endows cartilage with its tensile strength, whereas aggrecan, by virtue of its high negative charge, draws water into the tissue, swelling against the collagen network, thus enabling the tissue to bear loads and resist compression. Quantitatively more minor components (for example, type IX, XI, and VI collagens, biglycan, decorin, and cartilage oligomeric matrix protein) also have important roles in controlling matrix structure and organisation [2]. A healthy cartilage ECM is in a state of dynamic equilibrium, with a balance between synthesis and degradation. In the arthritides, this balance is disrupted and ECM degradation exceeds synthesis, resulting in a net loss of articular cartilage and underlying bone. The main enzymes responsible for this destruction are metalloproteinases, specifically a group of proteinases with a disintegrin and metalloproteinase domain with thrombospondin motifs (ADAMTS) and the matrix metalloproteinases (MMPs).

The aggrecanases (ADAMTS-1, -4, -5, -8, -9, and -15) cleave the interglobular domain separating the G1 and G2 domains of aggrecan specifically at the Glu^373^-Ala^374 ^bond [3,4], whereas MMPs can also cleave aggrecan at the nearby Asn^341^-Phe^342 ^bond. Aggrecanolysis involves both MMPs and aggrecanases; however, aggrecanase-mediated cleavage of aggrecan plays the major role in arthritis [5]. Recent compelling data from mouse knockout studies indicate that ADAMTS-5 is a key pathophysiological mediator of aggrecan catabolism in cartilage [6,7].

The human MMPs are a family of 23 enzymes that facilitate turnover and breakdown of the ECM in both physiology and pathology. MMPs are divided into several groups: collagenases, gelatinases, stromelysins, membrane-type MMPs, and glycosylphosphatidylinositol-anchored enzymes [8].

All metalloproteinases are synthesised in a latent form that requires the proteolytic removal of a pro-domain to generate the active enzyme. Metalloproteinase activity can be inhibited by tissue inhibitors of metalloproteinases (TIMPs), an endogenous family of four specific metalloproteinase inhibitors [9]. TIMPs have been shown to effectively block collagenolysis [10], indicating a role for metalloproteinases in this process, and TIMP-3 has been demonstrated to block aggrecanolysis [11], presumably via its ability to inhibit ADAMTS-4 and -5 [12]. In addition, a membrane-anchored glycoprotein, reversion-inducing cysteine-rich protein with Kazal motifs (RECK), has been identified and shown to inhibit MMP-2, -9, and -14 activity [13,14]. Metalloproteinase activity can also be inhibited by the general proteinase inhibitor alpha 2 macroglobulin (α_2_M). Thus, metalloproteinase activity is regulated at multiple levels: gene expression, post-translational activation of zymogens, and inhibition of the active enzyme [15]. Degradation of the collagenous network is excessive in arthritis [16], and the collagenases (MMP-1, -8, and -13), MMP-14 [17], and the gelatinase MMP-2 [18] all specifically cleave fibrillar collagen into characteristic three-fourth- and one-fourth-length fragments. This makes these enzymes key in the process of cartilage collagen turnover.

The cytokine combination of interleukin-1 (IL-1) + oncostatin M (OSM) synergistically induces the synthesis and activation of pro-collagenases, causing almost complete resorption of human and bovine nasal cartilage in a short assay period [19,20]. Natural and synthetic metalloproteinase inhibitors can prevent IL-1+OSM-induced cartilage destruction [10,21]. Bovine nasal cartilage is readily available, and this explant culture system provides a rapid, reproducible, and reliable model system to study the mechanisms of cartilage degradation and as such has become a standard for studying the efficacy of novel therapeutics (for example, [22,23]). Both IL-1 and OSM are relevant to joint destruction: increased levels of these cytokines are present in the arthritic joint [20,24], and adenoviral gene transfer of IL-1+OSM induces MMPs and joint damage in murine joints reminiscent to that seen in patients with rheumatoid arthritis (RA) [25].

The ECM not only provides physical support for cells but has now been shown to contain cryptic information that is released by metalloproteinases (reviewed in [26]). Metalloproteinases can liberate bioactive fragments from ECM macromolecules, release growth factors and cytokines embedded within the ECM, and cleave molecules present at the chondrocyte-ECM interface; all these can influence cellular behaviour. Thus, interactions between chondrocytes and their matrix are significant, so it is important to study these cells in their native environment. Many studies have looked at metalloproteinase regulation in chondrocytes grown in isolated monolayers (for example, [27,28]). However, there are very few studies on metalloproteinase expression and regulation in actively resorbing cartilage. The aim of this study, therefore, was to use an established model of active cartilage resorption to compare the temporal expression of metalloproteinases and their inhibitors and correlate this with pro-collagenase activation and aggrecan and collagen release.

## Materials and methods

### Cartilage degradation assay

Bovine nasal cartilage was cultured as previously described [20]. Briefly, bovine nasal septum cartilage was dissected into a diameter of approximately 2 mm by chips of 1- to 2-mm thickness. The cartilage was dispensed into tissue-culture flasks (0.7 g/flask) and incubated overnight in control, serum-free medium (Dulbecco's modified Eagle's medium containing 25 mM HEPES, 2 mM glutamine, 100 μg/ml streptomycin, 100 IU/ml penicillin, 2.5 μg/ml gentamicin, and 40 u/ml nystatin). Fresh control medium (10 ml) with or without IL-1+OSM (1 and 10 ng/ml, respectively) (in triplicate) was then added (day 0). At day 7, culture supernatants were harvested and replaced with fresh medium containing the same test reagents as day 0. Cartilage and culture supernatants were harvested in triplicate at days 0, 1, 3, 5, 7, 8, 10, 12, and 14. Hydroxyproline release was assayed as a measure of collagen degradation, and glycosaminoglycan release was assayed as a measure of proteoglycan degradation [20]. Collagenase and inhibitor activities in the culture supernatants were determined by the ^3^H-acetylated collagen diffuse fibril assay using a 96-well plate modification [29]. Aminophenylmercuric acetate (0.67 mM) was used to activate pro-collagenases. Inhibitory activity was assayed by the addition of samples to a known amount of active collagenase in the diffuse fibril assay. One unit of collagenase activity degrades 1 μg of collagen per minute at 37°C, and one unit of inhibitory activity inhibits two units of collagenase by 50%. Gelatinase activity in the culture supernatants was assayed by gelatin zymography. Samples were electrophoresed under non-reducing conditions by SDS-PAGE in 7.5% polyacrylamide gels copolymerised with 1% (wt/vol) gelatin. Gels were washed twice for 1 hour in 20 mM TrisHCl pH 7.8, 2.5% (vol/vol) Triton X-100 to remove SDS, then incubated 16 hours in 20 mM TrisHCl, pH 7.8, 10 mM CaCl_2_, 5 μM ZnCl_2_, and 1% (vol/vol) Triton X-100 at 37°C. Gels were then stained with Coomassie Brilliant Blue. Parallel gels were incubated in buffers containing 1,10-phenanthroline (2 mM) to show that lysis of gelatin was due to metalloproteinase activity.

### RNA extraction from cartilage

RNA was extracted from control and IL-1+OSM-stimulated cartilage at days 0, 1, 3, 5, 7, 8, 9, 10, 12, and 14. Cartilage was snap-frozen in liquid nitrogen. Immediately, this cartilage was ground for five cycles of 2 minutes of grinding and 2 minutes of cooling, in liquid nitrogen, at an impact frequency of 10 Hz in a SPEX CertiPrep 6750 freezer mill (Glen Creston, Stanmore, UK). Total RNA was isolated from the powdered cartilage essentially as described [30]. The cartilage was added to 5 ml TRIzol reagent (Invitrogen, Paisley, UK), shaken vigorously, and then centrifuged to remove insoluble material. Chloroform was added to the supernatant, and after centrifugation the aqueous phase was allowed to further separate for 2 days at 4°C. Afterward, the aqueous phase was mixed with a half volume of 100% ethanol and further purified using the RNeasy Mini kit, including an on-column DNAse I digestion (Qiagen, Crawley, UK).

### Real-time polymerase chain reaction

cDNA was synthesized from 1.0 μg of total RNA, using Superscript II reverse transcriptase and random hexamers in a total volume of 20 μl according to the manufacturer's instructions (Invitrogen). cDNA was stored at -20°C until used in downstream real-time polymerase chain reaction (PCR). Oligonucleotide primers were designed using DNAstar (DNASTAR, Inc., Madison, WI, USA) (Table 1). In 2004, the first assembly of the bovine genome sequence, with a 3.3-fold coverage, was deposited into free public DNA sequence databases, thus allowing the design of the metalloproteinase and inhibitor primer sets described. BLAST (Basic Local Alignment Search Tool) searches for all of the primer sequences were conducted to ensure gene specificity. Relative quantitation of genes was performed using the ABI Prism 7900HT sequence detection system (Applied Biosystems (Foster City, CA, USA). Metalloproteinase and inhibitor expression were determined using SYBR Green (Takara Bio Inc., Shiga, Japan) in accordance with the manufacturer's suggested protocol. PCR mixtures contained 50% Sybr-Green PCR mix (Takara Bio Inc.) and 100 nM of each primer in a total volume of 25 μl. Conditions for PCR were as follows: 10 seconds at 95°C, then 40 cycles each consisting of 5 seconds at 95°C, 15 seconds at 55°C, and 20 seconds at 72°C, followed by a dissociation plot. To confirm that the amplification produce was a single amplicon, products were analysed by agarose gel electrophoresis. The 18S rRNA gene was used as an endogenous control to normalise for differences in the amount of total RNA present in each sample; 18S rRNA TaqMan primers and probe were purchased from Applied Biosystems. TaqMan mastermix reagents (Sigma-Aldrich, St. Louis, MO, USA) were used according to the manufacturer's protocol.

Where data are presented as heat maps, the 2^-(CTgene-CT18S) ^(2^-ΔCT^) was used as an approximate measure of expression to allow comparison of expression levels between genes because it has been shown to correlate well between copy number (as assessed by using *in vitro*-transcribed RNA to produce a standard curve) and cycle threshold (C_T_) values [30]. The representation of 2^-ΔCT ^is therefore a useful means for the visualisation of multiple data sets.

## Results

### ADAMTS aggrecanases are differentially regulated during cartilage resorption

**Figure 1:**
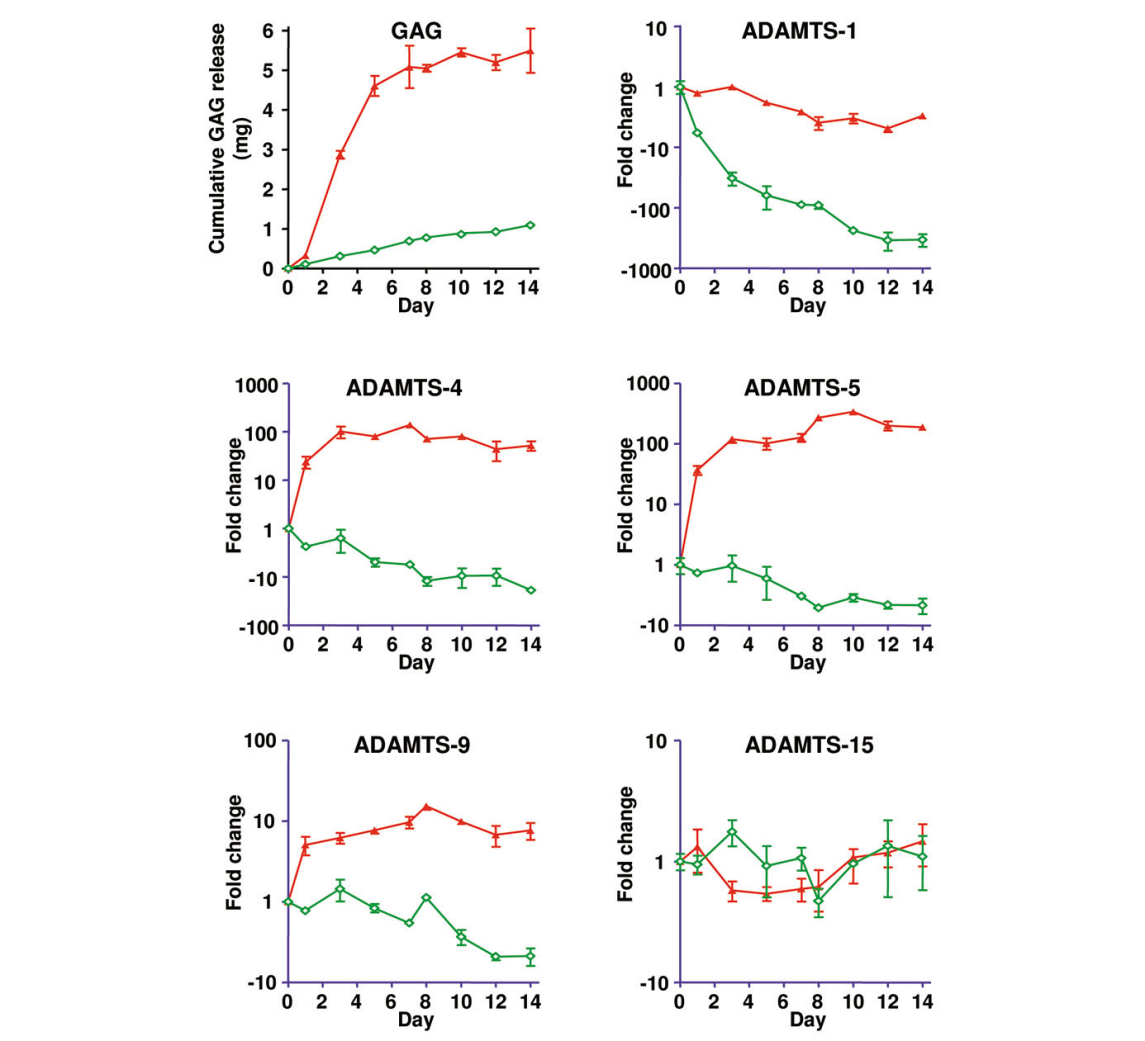
Profiling aggrecanase gene expression relative to aggrecanolysis in resorbing cartilage. Bovine nasal cartilage chips were cultured in medium ± IL-1+OSM (1 and 10 ng/ml, respectively) for 14 days. At day 7, medium was removed and the cartilage replenished with identical reagents. Cartilage and medium were harvested at days 0, 1, 3, 5, 7, 8, 10, 12, and 14. Each time point and condition were performed in triplicate. As a measure of proteoglycan, the levels of GAG released into the media from unstimulated (control) and IL-1+OSM-stimulated cartilage were assayed; cumulative GAG release is shown (*n *= 3). RNA was extracted from the cartilage, and *ADAMTS-1, -4, -5, -9*, and -*15 *gene expression was determined by real-time polymerase chain reaction (*n *= 3) as described in Materials and methods. The data are presented relative to *18S*. Values are the mean ± standard error of the mean. ◇ = control; ▲ = IL-1+OSM. ADAMTS, a disintegrin and metalloproteinase domain with thrombospondin motifs; GAG, glycosaminoglycan; IL-1, interleukin-1; OSM, oncostatin M.

By day 5 of culture, more than 80% of the proteoglycan was released from the cartilage stimulated with IL-1+OSM (Figure 1) in line with previous findings [14]. This was concomitant with the rapid and high levels of induction for *ADAMTS-4 *and *ADAMTS-5 *(100-fold) in the cartilage between days 0 and 3 of culture. *ADAMTS-9 *was also induced by IL-1+OSM but to a lower extent (10-fold). *ADAMTS-1 *was downregulated by IL-1+OSM during the culture compared with the basal expression at day 0 (fivefold), although IL-1+OSM stimulation results in higher *ADAMTS-1 *levels relative to control cartilage (>100-fold). *ADAMTS-15 *was detected at very low levels in cartilage but was not regulated, whereas *ADAMTS-8 *gene expression was undetectable.

### Multiple collagenases are expressed in resorbing cartilage

**Figure 2:**
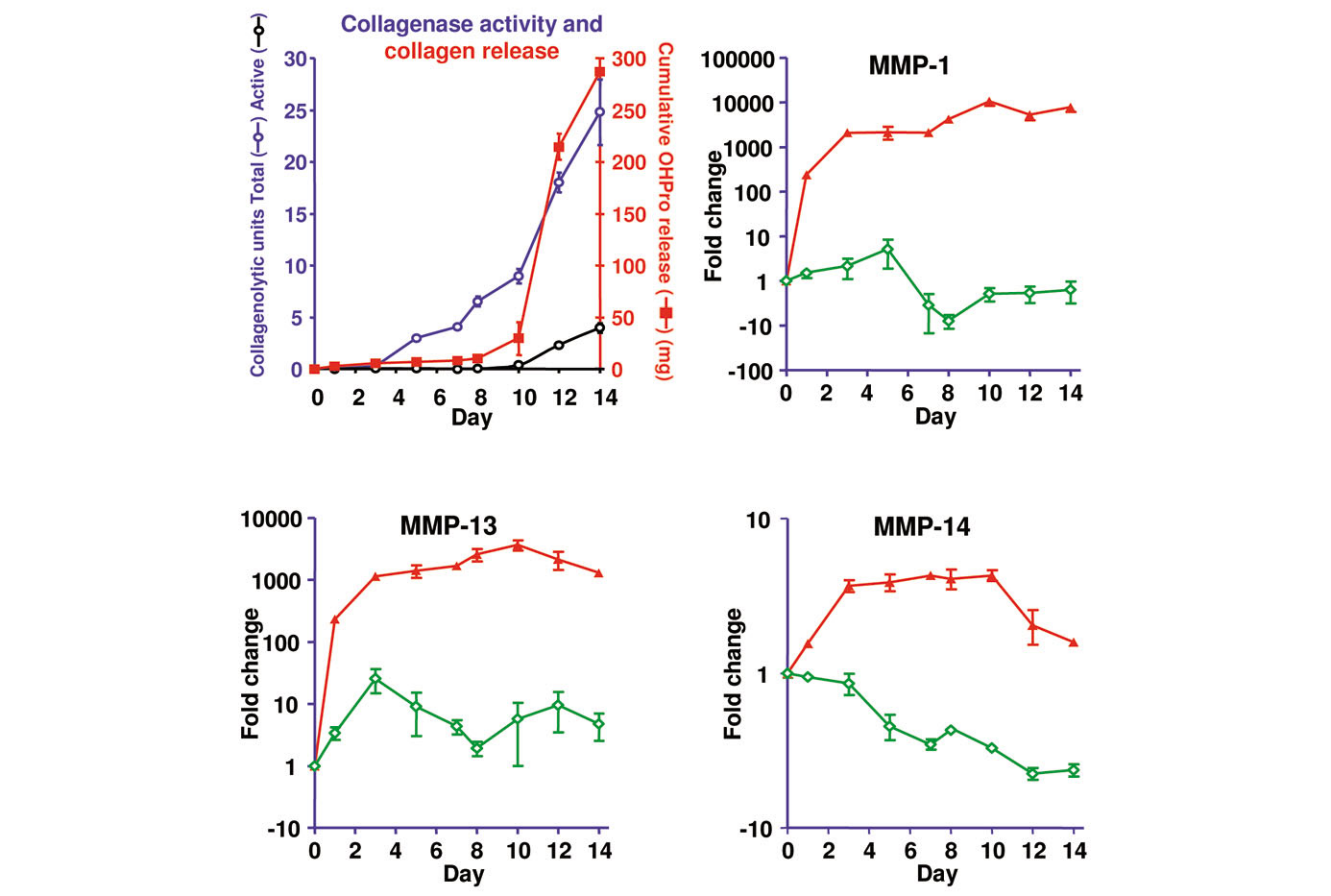
Profiling collagenase gene expression, collagenase activity, and collagenolysis in resorbing cartilage. Bovine nasal cartilage chips were cultured in medium ± interleukin-1 (IL-1) + oncostatin M (OSM) for 14 days exactly as described in the legend to Figure 1. As a measure of collagen, the levels of hydroxyproline (OHPro) released into the media from unstimulated (control) and IL-1+OSM-stimulated cartilage were assayed (*n *= 3); cumulative OHPro release is shown. Active collagenase activity in the media was assayed using the ^3^H-acetylated collagen diffuse fibril assay. Aminophenylmercuric acetate (0.67 mM) was used to activate pro-collagenases in order to measure the total collagenase activity (pro + active). RNA was extracted from cartilage, and matrix metalloproteinase (*MMP*) -*1*, -*13*, and -*14 *gene expression was determined by real-time polymerase chain reaction (*n *= 3) as described in Materials and methods. The data are presented relative to *18S*. Values are the mean ± standard error of the mean. ◇ = control; ▲ = IL-1+OSM.

Both *MMP-1 *(10,500-fold) and *MMP-13 *(3,700-fold) were rapidly and highly induced by IL-1+OSM in bovine nasal cartilage (Figure 2). Unlike *MMP-1 *and -*13, MMP-14 *had a high level of basal expression which was further induced by IL-1+OSM but to a lower extent (4-fold). *MMP-8 *could not be reproducibly detected in this assay. *MMP-1 *and -*13 *were rapidly induced early in the culture period, and pro-collagenases were first detected in the culture medium at day 5 (Figure 2). However, active collagenase and collagenolysis were not detected until day 10 of culture.

### MMP-9 is the predominant gelatinase in actively resorbing cartilage

**Figure 3:**
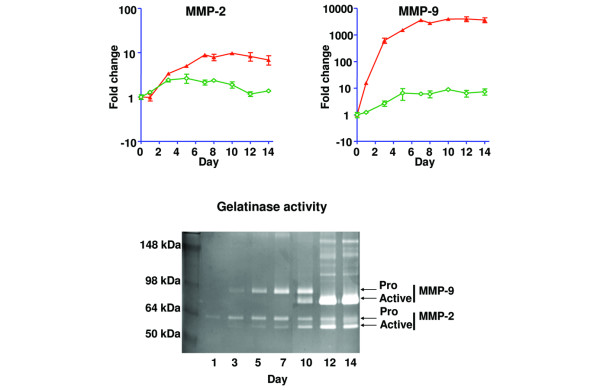
Profiling gelatinase gene expression and gelatinolytic activity in resorbing cartilage. Bovine nasal cartilage chips were cultured in medium ± interleukin-1 (IL-1) + oncostatin M (OSM) for 14 days exactly as described in the legend to Figure 1. RNA was extracted from cartilage, and matrix metalloproteinase (*MMP*)-*2 *and -*9 *gene expression determined by real-time polymerase chain reaction (*n *= 3) as described in Materials and methods. The data are presented relative to *18S*. As a measure of gelatinase activity, the culture media were analysed by gelatin zymography. Values are the mean ± standard error of the mean. ◇ = control; ▲ = IL-1+OSM.

The induction of *MMP-2 *was much slower and to a lower level (10-fold) than *MMP-9*, which was both rapidly and highly induced (4,000-fold) in the actively resorbing cartilage (Figure 3). ProMMP-9 (latent MMP-9) was first detected at day 3, but active MMP-9 was not present until day 10. The presence of pro and active forms of MMP-9 correlates with that of the collagenases (compare Figures 2 and 3), suggesting a similar activation mechanism for both proMMP-9 and the pro-collagenases. ProMMP-2 protein was constitutively expressed and active MMP-2 was first detected at day 3 in the cartilage medium, increasing thereafter.

### Non-collagenolytic MMPs are also regulated during cartilage resorption

**Figure 4:**
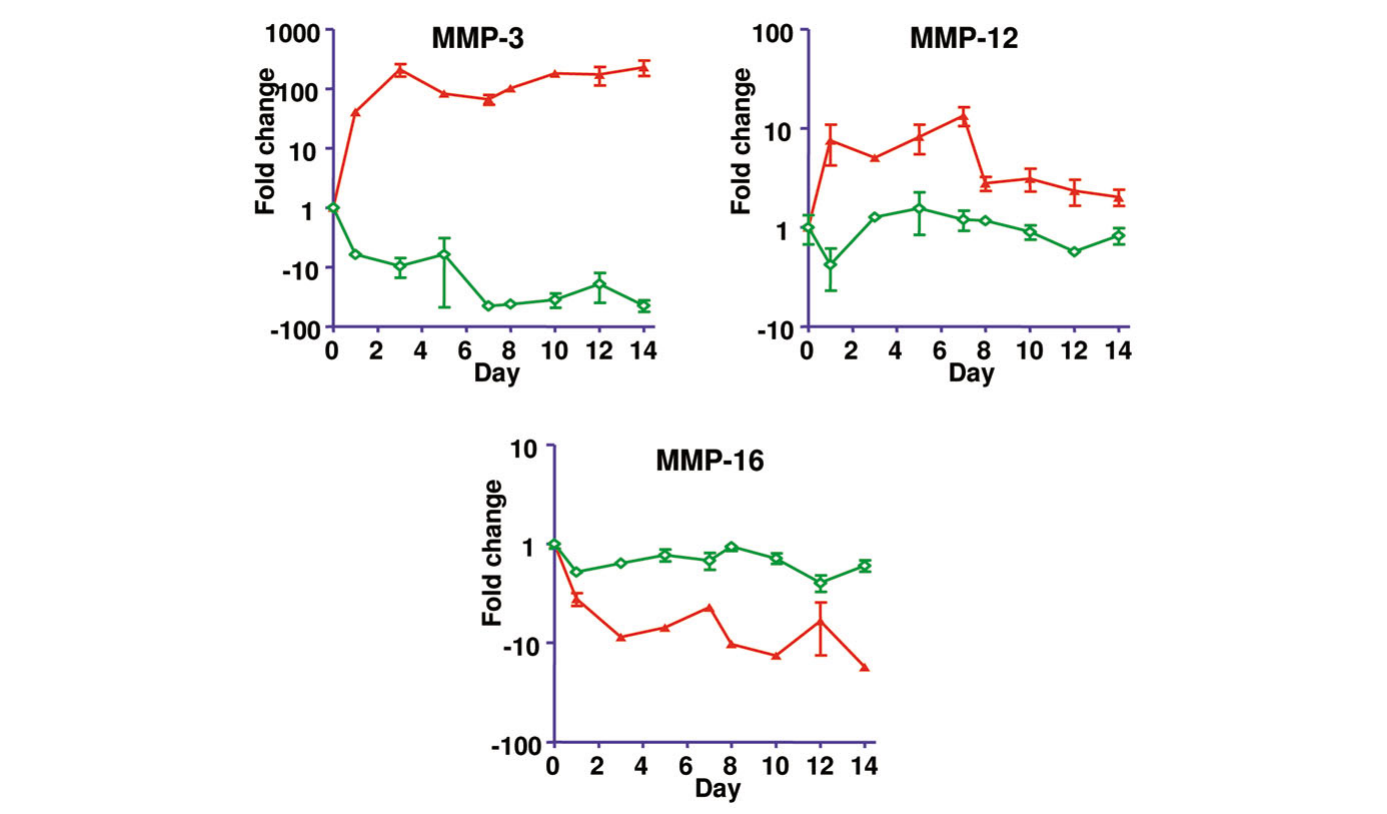
Profiling other matrix metalloproteinases (MMPs) in resorbing cartilage. Bovine nasal cartilage chips were cultured in medium ± interleukin-1 (IL-1) + oncostatin M (OSM) for 14 days exactly as described in the legend to Figure 1. RNA was extracted from cartilage, and *MMP-3, -12*, and -*16 *gene expression determined by real-time polymerase chain reaction (*n *= 3) as described in Materials and methods. The data are presented relative to *18S*. Values are the mean ± standard error of the mean. ◇ = control; ▲ = IL-1+OSM.

*MMP-3 *(stromelysin 1) basal expression was very low but was rapidly and highly induced in cartilage IL-1+OSM after stimulation (200-fold) (Figure 4), consistent with our previous observations in human articular chondrocytes [28]. *MMP-12 *(macrophage elastase) was also induced by IL-1+OSM but to a lower extent (<10-fold) (Figure 4). *MMP-16 (MT3-MMP) *was downregulated by IL-1+OSM in cartilage (10-fold) (Figure 4).

### Metalloproteinase inhibitor expression is downregulated during cartilage resorption

**Figure 5:**
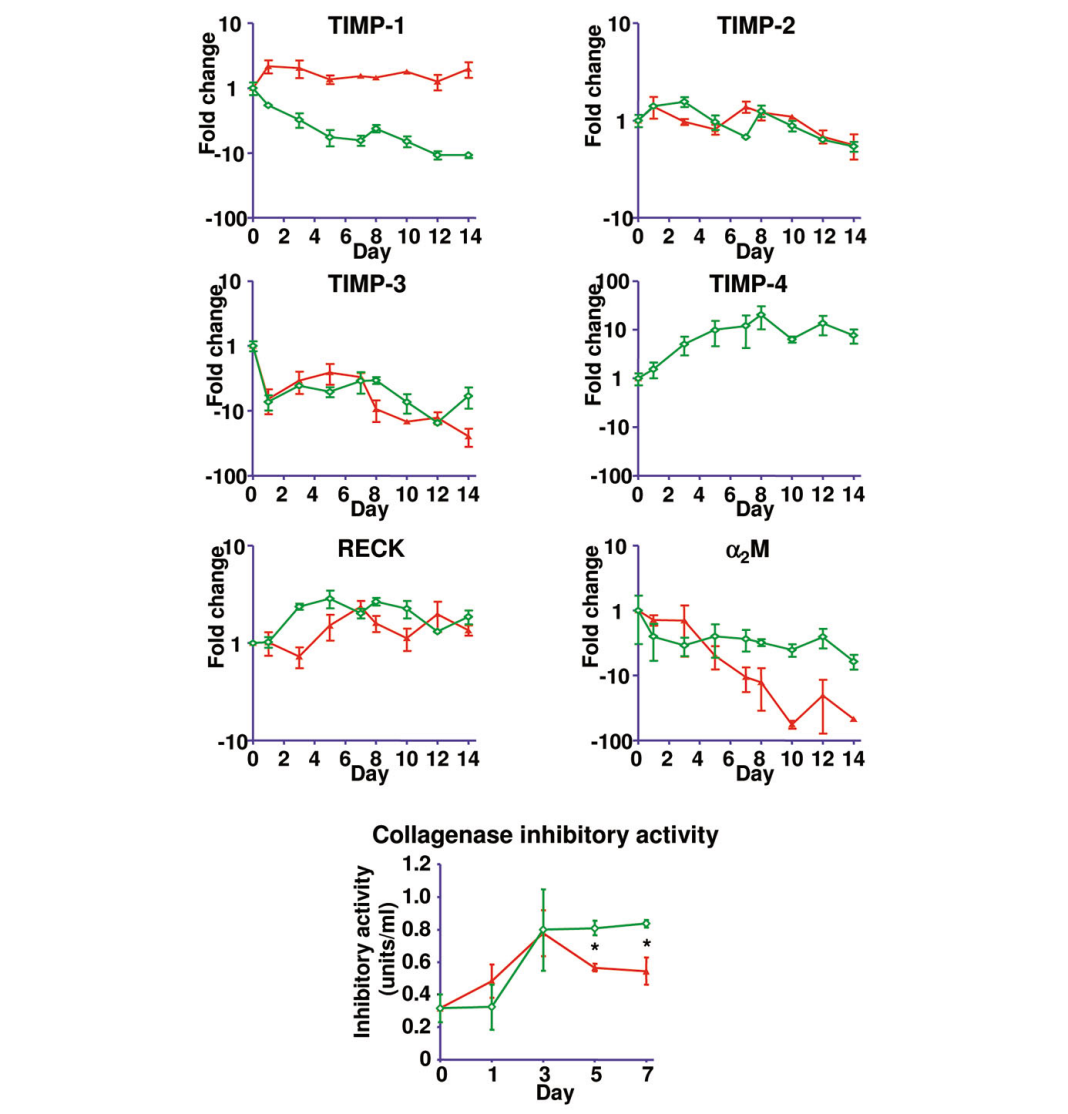
Profiling metalloproteinase inhibitor gene expression and inhibitory activity in resorbing cartilage. Bovine nasal cartilage chips were cultured in medium ± IL-1+OSM for 14 days exactly as described in the legend to Figure 1. RNA was extracted from cartilage, and *TIMP-1*, -*2*, -*3*, and -*4*, *RECK*, and α_2_*M *gene expression determined by real-time polymerase chain reaction (*n *= 3) as described in Materials and methods. The data are presented relative to *18S*. Inhibitory activity was assayed in the culture media by the addition of samples to a known amount of active matrix metalloproteinase-1 (MMP-1) in the diffuse fibril assay (*n *= 3). Values are the mean ± standard error of the mean. ◇ = control; ▲ = IL-1+OSM. *p < 0.05 using the Student's *t *test. α_2_M, alpha 2 macroglobulin; IL-1, interleukin-1; OSM, oncostatin M; RECK, reversion-inducing cysteine-rich protein with Kazal motifs; TIMP, tissue inhibitor of metalloproteinase.

A small induction of *TIMP-1 *(approximately twofold) after IL-1+OSM stimulation was seen in the cartilage, and although there was no clear regulation of either *TIMP-2 *or *TIMP-3 *by IL-1+OSM, there was a gradual reduction in expression levels during the culture period irrespective of the stimulation (Figure 5). *TIMP-4 *gene expression was detected in control cartilage and showed an increase (20-fold) in expression during the culture period. However, *TIMP-4 *was not detected in the IL-1+OSM-treated tissue. *RECK *was expressed at low levels by chondrocytes, but no regulation was observed during the culture, whereas α_2_*M *was downregulated (60-fold) in IL-1+OSM-treated cartilage (Figure 5). Inhibitory activity accumulated in the control culture media throughout the assay. However, IL-1+OSM conditioned media showed a sustained and significant decline of inhibitory activity from day 5. Due to the presence of active collagenase(s) and gelatinases, no inhibitory activity was detected in IL-1+OSM media after day 10 (Figures 2 and 3).

### Gene expression analysis reveals the differential levels of metalloproteinase and inhibitor transcripts during cartilage homeostasis and resorption

**Figure 6:**
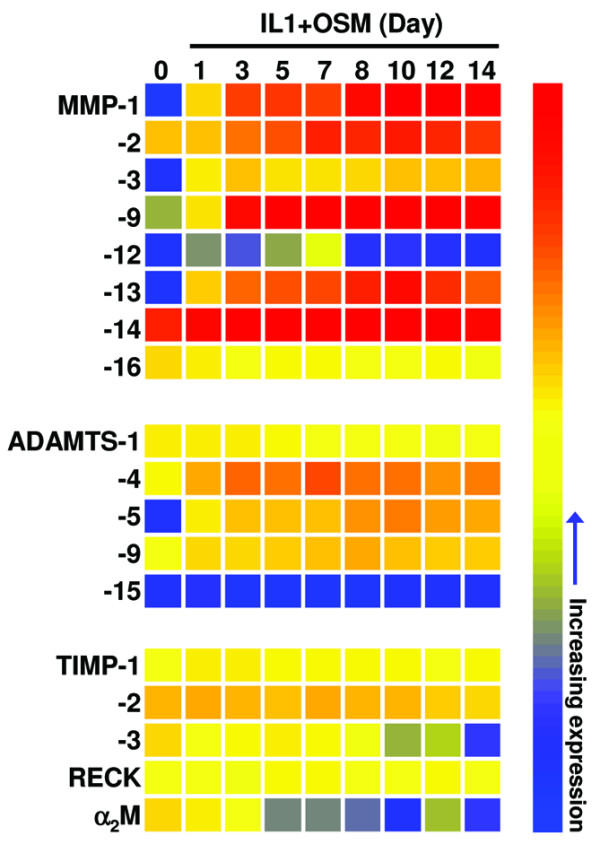
Relative differential expression of MMPs, ADAMTS, and metalloproteinase inhibitors in resorbing cartilage. Bovine nasal cartilage chips were cultured in medium ± IL-1+OSM for 14 days exactly as described in the legend to Figure 1. RNA was extracted from the cartilage, and metalloproteinase and inhibitor gene expression were determined by real-time polymerase chain reaction as described in Materials and methods. The mean 2^-ΔCT ^of each gene (where ΔC_T _is calculated as [C_T _gene - C_T _18S]) was used as a measure of relative gene expression to allow simultaneous comparisons. The heat map was generated using GeneSpring GX 7.3 (Agilent Technologies, Palo Alto, CA, USA) with the expression range set at 0.025 (high), 5 × 10^-6 ^(normal), and 1 × 10^-10 ^(low) arbitrary units. ADAMTS, a disintegrin and metalloproteinase domain with thrombospondin motifs; C_T_, cycle threshold; IL-1, interleukin-1; MMP, matrix metalloproteinase; OSM, oncostatin M.

Using the comparative C_T _method (2^-ΔCT^), we compared the mean relative expression levels of all the genes before and during the resorptive process (Figure 6). At day 0, the chondrocytes expressed little *MMP-1 *or -*13 *whereas *MMP-14 *was the most abundant transcript detected. Of the potential aggrecanases, *ADAMTS-5 *and -*15 *showed the lowest expression at day 0, and of these only *ADAMTS-5 *increased during resorption. The metalloproteinase inhibitors were all relatively abundant at day 0, but overall these levels decreased during the resorptive process.

## Discussion

The stimulation of bovine nasal cartilage with IL-1+OSM represents a rapid and reproducible model of the cartilage destruction that is prevalent in the arthritides [20]. This model is a useful assay system for studying the mechanisms of cartilage degradation (for example, [31,32]) and the efficacy of novel therapeutics (for example, [22,23,33,34]). Several studies have profiled the expression and regulation of metalloproteinases and their inhibitors in response to pro-inflammatory cytokines in chondrocytes [27,28]; however, these studies have been confined to investigating gene expression in isolated chondrocyte monolayers. Here, we have profiled for the first time the gene expression of multiple metalloproteinases and their inhibitors in actively resorbing cartilage by real-time PCR. Furthermore, we have correlated this gene expression with gelatinase and collagenase enzyme expression and activation, as well as proteoglycan and collagen release.

Our data suggest that in the bovine model ADAMTS-4, -5, and -9, but not ADAMTS-1, -8, and -15, could be important enzymes associated with aggrecanolysis. Previous studies have investigated the regulation of *ADAMTS-4 *and -*5 *at a single time point in IL-1-treated cartilage explant cultures and indicate that IL-1 upregulated these aggrecanases in bovine articular cartilage cultured for 4 days [35,36] or 1 day [37] and that IL-1 increased *ADAMTS-4 *and -*5 *in murine cartilage cultured for 3 days [7]. Conversely, IL-1 upregulated *ADAMTS-4 *in bovine articular cartilage cultured for 3 days whereas *ADAMTS-5 *was constitutively expressed [38]. Tortorella *et al. *[38] used a semi-quantitative PCR technique, which may explain the discrepancies in the results compared with our study in which real-time PCR was used. The regulation of *ADAMTS-1*, -*8*, -*9*, and -*15 *in cartilage explant cultures has not been previously reported.

Our observations of the upregulation of *ADAMTS-4*, -*5*, and -*9 *by IL-1+OSM in cartilage explants are consistent with our previous studies of chondrocyte monolayers in which IL-1+OSM upregulated these *ADAMTS *genes in primary human articular chondrocytes [39] and *ADAMTS-4 *and -*5 *in a human chondrocyte cell line [27]. We have previously shown that *ADAMTS-1 *was only weakly induced in response to IL-1+OSM in a human chondrocyte cell line [27] and shows no change in monolayer cultured human articular chondrocytes (JB Catterall, unpublished data). Data from IL-1+OSM-stimulated human articular chondrocyte monolayers show that *ADAMTS-8 *was not detectable [40] and *ADAMTS-15 *was downregulated (JB Catterall, unpublished data), consistent with and further supporting our findings in actively resorbing bovine nasal cartilage explants. The basal (day 0) relative expression levels of the ADAMTSs further suggest that ADAMTS-4 and -9 may be important for cartilage homeostasis and support the hypothesis that in the bovine system, as in murine arthritis, ADAMTS-5 may be critically important [6,7].

Although basal expression of *MMP-3 *(stromelysin 1) and *MMP-12 *(macrophage elastase) was very low, both were induced in cartilage after IL-1+OSM stimulation. MMP-3 is an activator of several proMMPs, including the collagenases proMMP-1 [41], proMMP-8 [42], and proMMP-13 [43], and thus may have an important role in the cascades leading to cartilage collagenolysis. Indeed, we have previously shown that exogenous addition of MMP-3 to cartilage can mediate pro-collagenase activation and effect such collagenolysis [21,44]. Over-expression of *MMP-12 *has been shown to enhance the development of inflammatory arthritis in transgenic rabbits [45], and there is increased expression of MMP-12 in RA synovial tissues compared with osteoarthritis (OA) [46], suggesting that MMP-12 may play a destructive role in arthritis. Like *MMP-3*, the relatively early *MMP-12 *induction suggests that it may be involved in the proteolytic events that occur after aggrecanolysis and prior to collagenolysis. The downregulation of *MMP-16 (MT3-MMP) *by IL-1+OSM in cartilage implies that this membrane-bound MMP is not involved in the cascades leading to cartilage degradation. IL-1 and/or OSM also failed to clearly modulate *MMP-16 *expression in a human chondrocyte cell line [27], and a role of MMP-16 in arthritis remains unclear although it is expressed in rheumatoid synovium [47] and is elevated in end-stage OA compared with normal cartilage [30].

The collagenase expression data are consistent with our previous studies that show IL-1+OSM upregulates *MMP-1*, -*13*, and -*14 *in primary human articular chondrocytes and chondrocyte cell lines [25,27,28]. *MMP-8 *could not be reproducibly detected in this assay. However, we have shown that *MMP-8 *is induced at low levels by IL-1+OSM in a human chondrocyte cell line [27] and in bovine nasal and human articular chondrocytes [25]. Thus, the key collagenases involved in IL-1+OSM-induced cartilage collagenolysis are likely to be MMP-1 and/or -13. The rapid induction of these collagenase genes early after IL-1+OSM stimulation was surprising considering that, as with our previous results using this model [21], pro-collagenases were not detected in the culture medium until day 5 and active collagenase and collagenolysis were not detected until day 10. Thus, activation of pro-collagenases appears to be delayed relative to collagenase expression, and this step is a key control point that dictates whether cartilage collagen degradation will occur. Previous studies in other matrices such as periosteal tissue have shown that a large amount of proMMP-1 is stored and only when activated results in complete breakdown of this collagenous ECM [48], thus supporting the central importance of pro-collagenase activation in ECM breakdown. Furthermore, our observations are consistent with our previous studies that showed that either a furin-like enzyme inhibitor, Dec-RVKR-CH_2_Cl, or the general trypsin-like serine proteinase inhibitor, alpha1-proteinase inhibitor, can block the activation of pro-collagenases and degradation of collagen in the bovine nasal cartilage assay [21,49]. Also, the kinetics of proMMP-9 and collagenase activation appears similar, suggesting their activation is via the same serine protease-dependent cascade. ProMMP-2 was activated at an earlier point in the cartilage assay, when collagenolysis was absent, implying that this gelatinase is unlikely to be a key collagenase with respect to cartilage collagenolysis. This early activation also suggests an alternative activation mechanism to that of either proMMP-9 or the pro-collagenases. ProMMP-2, but not proMMP-9, can be activated by MMP-14 [50], which was highly abundant throughout the assay and can itself be processed by furin [51]. Interestingly, though MMP-2-deficient mice are viable, MMP-14-deficient mice show an impairment of cartilage resorption during endochondral ossification, and therefore pro-MMP-2 activation is probably not the only role of MMP-14 in cartilage or mice *per se *[52]. Taken together, these data suggest that serine proteinases are involved in the activation cascades of the pro-collagenases and pro-gelatinases that result in cartilage resorption [21,49].

Because cartilage resorption induced by IL-1+OSM can be prevented by the addition of exogenous TIMPs [10], it was important to monitor metalloproteinase inhibitor expression during this resorption. Of the TIMPs, only *TIMP-1 *was induced after IL-1+OSM stimulation, consistent with our previous studies that showed a transient induction of TIMP-1 by IL-1+OSM in bovine and human chondrocyte monolayers [25]. Both *TIMP-2 *and *TIMP-3 *expression gradually decreased during the assay even with IL-1+OSM, and *TIMP-4 *expression was detected in control cartilage only. Furthermore, the general proteinase inhibitor α_2_*M *was also downregulated in the assay, consistent with the observation that α_2_*M *is downregulated in IL-1-treated primary human articular chondrocytes [53]. *RECK *was expressed at low levels and was not regulated. Thus, there was an overall reduction in the levels of free inhibitory activity in cartilage after IL-1+OSM stimulation which was evident by day 5, probably due to the increase in active metalloproteinase levels. This suggests that activation of proMMPs occurs as early as day 5 of culture. Although the inhibitory bioassay used does not discriminate between TIMPs and other metalloproteinase inhibitors, the reduction in inhibitory activity between days 7 and 14 correlates well with the reduction in the observed mRNA levels for *TIMP-2*, -*3*, and -*4 *and α_2_*M*. The combination of an overall decrease in inhibitor gene expression, coupled with the dramatically increased expression of specific metalloproteinases and their subsequent activation, results in a net shift in the TIMP-metalloproteinase balance favouring the metalloproteinases and hence cartilage destruction.

## Conclusion

This is the first study to profile the expression of multiple metalloproteinases and their inhibitors in actively resorbing cartilage. *MMP-1*, -*2*, -*3*, -*9*, -*12*, -*13*, and -*14 *and *ADAMTS-4*, -*5*, and -*9 *gene expression was induced in bovine nasal cartilage explants stimulated to resorb with IL-1+OSM. These enzymes represent a potent combination of proteinases that contribute to the proteolytic mechanisms resulting in cartilage degradation. All were markedly upregulated in the first few days after stimulation and, although pro-collagenases were also detected early, active collagenase(s) and collagenolysis were not detected until day 10 of culture. IL-1+OSM also causes a net reduction in metalloproteinase inhibitors, favouring the destructive potential of the plethora of metalloproteinases that this potent cytokine combination induces. The abundant expression of MMP-14 throughout the assay, along with phenotypic analysis of MMP-14-deficient mice [52], suggests a role for this enzyme in cartilage homeostasis.

We have previously shown that there is sufficient pro-collagenase early in the cartilage culture which, if activated, leads to cartilage collagen resorption [21]. Thus, activation of pro-collagenases is a key control point in the breakdown of the cartilage collagen matrix.

The observations described in this study corroborate our previous data in human chondrocyte monolayer cultures that have shown that IL-1+OSM markedly upregulates several metalloproteinases [20,27,28]. We have also shown that human nasal cartilage responds to IL-1+OSM with the synergistic induction of MMP-1, MMP-13, collagenolytic activity, and collagenolysis [19], thus further validating the bovine nasal cartilage degradation assay as a reliable and useful model to study human disease. Indeed, it is highly applicable for studying the mechanisms of cartilage degradation such as activation of pro-collagenases, which represents an important potential target for intervention therapies that prevent the tissue destruction prevalent in arthritis.

## Abbreviations

ADAMTS = a disintegrin and metalloproteinase domain with thrombospondin motifs; α_2_M = alpha 2 macroglobulin; C_T _= cycle threshold; ECM = extracellular matrix; IL-1 = interleukin-1; MMP = matrix metalloproteinase; OA= osteoarthritis; OSM = oncostatin M; PCR = polymerase chain reaction; ProMMP = Prdomain containing (i.e. latent) matrix metalloproteinase; RA = rheumatoid arthritis; RECK = reversion-inducing cysteine-rich protein with Kazal motifs; TIMP = tissue inhibitor of metalloproteinase.

## Competing interests

The authors declare that they have no competing interests.

## Authors' contributions

JMM helped extract RNA from cartilage, performed the cartilage assays, and helped conceive, design, and coordinate the study and draft the manuscript. ADR and TEC helped conceive, design, and coordinate the study and draft the manuscript. DAY helped extract RNA from cartilage, designed PCR primers, performed the real-time PCR, conceived, designed, and coordinated the study, and drafted the manuscript. All authors read and approved the final manuscript.

## Figures and Tables

**Table 1:** Bovine metalloproteinase and inhibitor primers for real-time polymerase chain reaction

Gene	Sequence (5'-3')	Length (bp)
*MMP-1*	GATGCCGCTGTTTCTGAGGA	372
	GACTGAGCGACTAACACGACACAT	
*MMP-2*	TCTGCCCCCATGAAGCCCTGTT	347
	GCCCCACTTGCGGTCATCATCGTA	
*MMP-3*	TTAGAGAACATGGGGACTTTTTG	360
	CGGGTTCGGGAGGCACAG	
*MMP-8*	ATGCTGCTTATGAGGATTTTGACA	101
	GCCTGGGGTAACCTTGCTGAGTA	
*MMP-9*	CGCCACCACCGCCAACTACG	350
	GGGGGTGCTCCTCTGTGAATCTGT	
*MMP-12*	TGTGACCCCAATATGAGTTTT	155
	TTGAATGTAAGACGGTAGGTTT	
*MMP-13*	CCCTCTGGTCTGTTGGCTCAC	304
	CTGGCGTTTTGGGATGTTTAGA	
*MMP-14*	AGGCCGACATCATGATCTTCTTTG	375
	CTGGGTTGAGGGGGCATCTTAGTG	
*MMP-16*	ACCCCAGGATGTCAGTGC	287
	AATAGCTTTACGGGTTTCAGG	
*TIMP-1*	TGGGCACCTGCACATCACC	277
	CATCTGGGCCCGCAAGGACTG	
*TIMP-2*	ATAGTGATCAGGGCCAAAGCAGTC	277
	TGTCCCAGGGCACGATGAAGTC	
*TIMP-3*	GATGTACCGAGGATTCACCAAGAT	356
	GCCGGATGCAAGCGTAGT	
*TIMP-4*	ATATTTATACGCCTTTTGATTCCT	297
	GGTACCCGTAGAGCTTCCGTTCC	
α_2_*M*	GCCCGCTTTGCCCCTAACA	359
	TCGTCCACCCCACCCTTGATG	
*RECK*	GTAATTGCCAAAAAGTGAAA	352
	TAGGTGCATATAAACAAGAAGTA	
*ADAMTS-1*	GCTGCCCTCACACTGCGGAAC	264
	CATCATGGGGCATGTTAAACAC	
*ADAMTS-4*	GCGCCCGCTTCATCACTG	101
	TTGCCGGGGAAGGTCACG	
*ADAMTS-5*	AAGCTGCCGGCCGTGGAAGGAA	196
	TGGGTTATTGCAGTGGCGGTAGG	
*ADAMTS-8*	AGATCTTTGGGCTGGGCTTCC	116
	GGCTGGCATTCCTCGTGTGG	
*ADAMTS-9*	GGGAGCGGAAACGAAAACCTATT	167
	CACTGGGCACTACATTCACTCCTG	
*ADAMTS-15*	GACACGGCCATCCTCTTCACTCG	107
	AGCAGCTCCTCTTGGGGTCACAC	

ADAMTS, a disintegrin and metalloproteinase domain with thrombospondin motifs; α_2_M, alpha 2 macroglobulin; MMP, matrix metalloproteinase; RECK, reversion-inducing cysteine-rich protein with Kazal motifs; TIMP, tissue inhibitor of metalloproteinase.
